# Fish Bone-Induced Pancreatitis

**DOI:** 10.7759/cureus.26191

**Published:** 2022-06-22

**Authors:** Neha Sharma, Kaveh Zivari, Avleen Kaur, Amina Kureshi, Ira Mayer

**Affiliations:** 1 Internal Medicine, Maimonides Medical Center, New York City, USA; 2 Gastroenterology, Maimonides Medical Center, New York City, USA; 3 Gastroenterology, New York Institute of Technology (NYIT) College of Osteopathic Medicine, Old Westbury, USA

**Keywords:** foreign body pancreatitis, upper endoscopy, fish bone induced pancreatitis, pancreatitis, foreign body retrieval

## Abstract

Fish bone-induced pancreatitis is an uncommon cause of pancreatitis, with only a few reported cases in the literature. The patients with the highest risk for fish bone-induced pancreatitis include those from cultures where unfilleted fish is a culinary delicacy. The etiology of foreign body-induced pancreatitis is very common, secondary to inflammation of the duodenal papilla or bile duct obstruction. CT imaging is key for visualization of the fish bone, as radiography rarely detects fish bones. Complications of fish bone-induced pancreatitis include thrombosis of the superior mesenteric vein, bacteremia (with *Peptostreptococcus*), pancreatic granuloma, and gastrointestinal perforation. Management of fish bone-induced pancreatitis includes either endoscopic resection or exploratory laparotomy, followed by supportive care until pancreatitis resolves. Here, we present a case of pancreatitis secondary to accidental fish bone ingestion, identified during upper gastrointestinal endoscopy and managed by bone removal and supportive care.

## Introduction

Foreign body-induced pancreatitis is a relatively known cause of pancreatitis. However, fish bone-induced pancreatitis is an exceedingly rare cause in this category. The cases reported thus far are mostly seen in cultures where unfilleted fish is a culinary delicacy. A high index of suspicion is required for diagnosis, as history is often unyielding. Imaging, particularly CT scan of the abdomen, is the gold standard for establishing the diagnosis. Prompt removal of the bone via upper GI endoscopy or exploratory laparotomy is the definitive treatment, along with supportive care for pancreatitis.

This article was previously presented as a meeting abstract at the 2021 American College of Gastroenterology (ACG) Annual Conference on October 24, 2021.

## Case presentation

**Figure 1:**
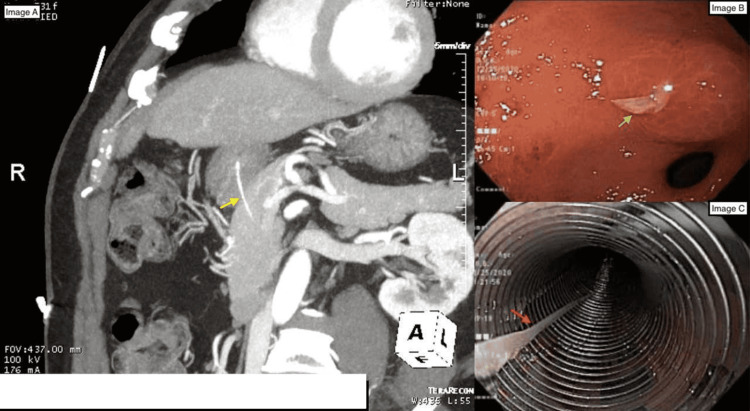
(A) Fish bone penetrating through stomach wall into the pancreas (yellow arrow); (B) One end of the fish bone seen protruding into the prepyloric region on upper GI endoscopy (green arrow); (C) Retrieved fish bone during endoscopy (red arrow).

A 64-year-old male, with past history of diabetes and chronic obstructive pulmonary disease (COPD), presented with epigastric pain for two days. The pain was sudden in onset, started at 3-4/10 on a 10-point pain scale, but escalated to 10/10 at the time of presentation, and was intermittent and non-radiating, associated with nausea. The patient could not identify any relationship between the pain and meals or position. In the systemic review, pertinent cardiac symptoms, particularly left-sided chest pain, palpitations, shortness of breath, or light-headedness, were negative. There was no history of alcohol use, gall bladder stones, recent abdominal instrumentation, or identified scorpion bite. On initial presentation the patient was normothermic, tachycardic, likely due to pain, and normotensive, with peripheral arterial saturation of 95% breathing ambient air. The patient had a tender abdomen upon palpation. Laboratory values were significant for leukocytosis to 17.4 K/L and lipase 512 U/L (reference range 8-69 U/L). CT-abdomen showed a 3.3 cm fish bone extending from the distal stomach into the head of the pancreas. The patient was admitted, made nil per oral, and was started on intravenous crystalloid fluids for pancreatitis while gastroenterology was consulted. The fish bone was removed via upper GI endoscopy after being seen protruding in the pre-pyloric area (Figure 1). The patient's leukocytosis slowly improved, lipase trended down, and pain resolved after fish bone removal and supportive care.

## Discussion

Foreign body ingestion is a common occurrence in children but also adults of all ages, mostly as a result of an accidental event. The cough reflex is a protective mechanism that functions to clear the respiratory tract of accidentally or otherwise aspirated foreign bodies. However, the GI tract is not equipped with any comparable mechanism. What GI tract has as a defense mechanism is the acidity of the stomach that acts to neutralize the microbes ingested accidentally. But many macroscopic foreign bodies, when ingested, can pass through this line of defense to result in complications downstream.

Fish bone is one of the most commonly ingested foreign bodies. However, less than 1% of the ingested foreign bodies result in bowel perforation [1]. The gut's anatomical constrictions are cricopharyngeal sphincter, esophageal constrictions (due to arch of aorta and bronchus), C loop of the duodenum distal ileum, and ileocecal junction, are the common anatomical sites of foreign body impaction. The most common perforation sites are the terminal ileum, sigmoid colon, and rectum, followed by the C-loop of the duodenum [2,3]. Rarely the bone can migrate through the gut wall to lodge into the adjacent organs and cause inflammation or abscess formation. Gastric or duodenal perforation and penetration of the pancreas resulting in pancreatitis, as seen in our case, is an exceedingly rare presentation of accidental fish bone ingestion. Some other complications include thrombosis of the superior mesenteric vein (if bone migrates to this vein), bacteremia (particularly with *Peptostreptococcus*, which is part of the normal flora of the GI tract), pancreatic granuloma, and gastrointestinal perforation, as a result of mechanical migration of the bone, regardless of the severity of pancreatitis.

It is difficult to establish the temporal relationship between fish bone ingestion and the symptoms produced by its penetration into the abdominal organs, as patients generally do not recall a clear history. Hence, to preoperatively diagnose this condition, a very high index of suspicion is needed. The symptoms can range from nondescript abdominal pain to pancreatitis-like upper abdominal pain that radiates to the back [2,4]. Lab values can show leukocytosis and elevated pancreatic amylase and lipase. The CT scan is the modality of choice for pre-operative diagnosis. The bone can be retrieved via upper endoscopy if the bone is amenable to endoscopic removal (as seen in our case). In cases where the bone is far advanced in the body of the pancreas, exploratory laparotomy should be considered.

## Conclusions

Foreign body-induced pancreatitis is a rare presentation of pancreatitis. Even a thorough history can miss the history of foreign body ingestion, especially foreign bodies associated with food ingestion. A high index of suspicion is required to diagnose this unique modality of pancreatitis. Once diagnosed, the treatment involves removal of the foreign body and supportive care.
